# Primary Immunodeficiencies and Oncological Risk: The Experience of the Children's Hospital of Brescia

**DOI:** 10.3389/fped.2019.00232

**Published:** 2019-06-19

**Authors:** Marianna Maffeis, Lucia Dora Notarangelo, Richard Fabian Schumacher, Elena Soncini, Annarosa Soresina, Arnalda Lanfranchi, Fulvio Porta

**Affiliations:** ^1^Pediatric Oncohematology and Bone Marrow Transplant (BMT) Unit, Children's Hospital, Spedali Civili, Brescia, Italy; ^2^Pediatric Immunology Unit, Department of Pediatrics, Children's Hospital, Spedali Civili, Brescia, Italy; ^3^Stem Cell Laboratory, Section of Hematology and Blood Coagulation, Diagnostic Department, ASST Spedali Civili, Brescia, Italy

**Keywords:** primary immumunodeficiencies, tumors, pediatric, bone marrow transplant (BMT), rare diseases

## Abstract

**Background and aims:** Primary immunodeficiencies (PID) are characterized by recurrent infections and increased risk of malignancies because of the reduced immunological surveillance against cancer cells and oncogenic viruses.

**Methods:** We report the incidence of tumors among 690 patients with PID, diagnosed from 1990 until 2017 in Brescia.

**Results:** Out of 690 patients, 25 patients (3.6%) developed 33 tumors. Of the 25 affected patients, 8 patients suffered from common variable immunodeficiency (CVID), 5 from combined immunodeficiency (CID), 3 from Ataxia-telangectasia (AT), 2 from Hermanksy-Pudlak type 2 (HSP2), 2 from gammaglobulinemia X-linked (XLA), 2 from Wiskott-Aldrich syndrome (WAS), 2 from Hyper IgE syndrome (HIES), 1 from severe combined immunodeficiency (SCID). The age at diagnosis ranged from 1 to 52 years, with a median age of 19.6 years. The time between the diagnosis of PID and onset of tumor was short, often <1 year between diagnosis and the appearance of cancer in the case of CID. Moreover, in two cases of CID, the diagnosis of cancer was made before the diagnosis of PID, so cancer was the onset clinical manifestation. Hematological malignancies were prevalent (22/33, 66.7%) with a minority of solid tumors (11/33, 33.33%). In particular Non-Hodgkin lymphomas were the most frequent (16/33, 48.48%). In total 13 patients survived (52%) and tumor was the main cause of death (7 cases). Two patients underwent BMT once the disease was in remission.

**Conclusions:** Therefore, the correct management of tumors that arise in patients with primitive immunodeficiency still represents a challenge in the pediatric field. For this reason now it is mandatory to collect in a unique international registry the cases of malignancies in PID that could lead to a better understanding of the etiopathogenesis and of the biological and clinical characteristics of these tumors, with the aim of defining adequate preventive measures and guaranteeing an early diagnosis which also creating a shared and specific therapeutic strategy, with the prospect of obtaining a better prognosis for these patients.

## Background and Aims

Primary immunodeficiencies (PID) represent a heterogeneous group of congenital diseases characterized by an alteration of the functionality of the immune system. These diseases are considered rare, in fact in Italy the incidence of these forms is variable, ranging from 1:500 for the most frequent forms to 1:500 000 for other rare forms. The overall incidence of PID can be calculated around 1:2000^1^. The classification of these diseases is very complex and constantly evolving in relation to genetic mutations and the etiopathogenetic mechanisms that are identified progressively over the years. Depending on the component of the immune system that is altered, they can be divided into two main groups: the deficiencies of innate immunity and the deficiencies of adaptive immunity. In particular, the latter group includes the deficits of humoral immunity, the combined immunity deficits, the immune deficiencies associated with syndromes, and the immune dysregulation diseases.

These diseases are characterized by an increased susceptibility to infections, by the frequent development of autoimmune diseases and by a predisposition toward the onset of neoplasia. This particular case became even more evident in recent years since the development of drugs for the prophylaxis and the treatment of opportunistic infections made it possible to have an increase of the life expectancy of these patients, providing the necessary time for neoplastic development. Indeed, tumors are currently the second leading cause of death for patients with PID after infections.

The high incidence of tumors is primarily evident in the combined immunodeficiencies in which there is a defect of cell-mediated immunity that plays a fundamental role in the control of birth and neoplastic growth, a mechanism that it is called Tumor Immunosurveillance (1–3). In fact, cytotoxic T lymphocytes are able to specifically recognize tumor cells and induce them to death. Moreover, the development of tumors also occurs as a consequence of the numerous infections affecting these patients: first of all the oncogenic viruses (Epstein-Barr virus, Human Papilloma Virus), which can directly cause a degeneration of the cells toward neoplastic forms; we also need to consider the chronicization of infections: by establishing a persistent inflammatory state, they create tissue damage, which can be precursor of a subsequent malignant transformation (such as for Helycobacter pilori) (4–6). Moreover, these mechanisms are joined by the tendency to acquire frequent genetic mutations, the result of genomic instability that characterizes some PID. This condition occurs in the following cases:

- presence of defects in DNA damage repair (Ataxia-telangectasia) (7)
- presence of defects of apoptosis that, causing cellular immortalization, allow cells to survive even in the presence of irreversible damage to the genome (Autoimmune Lymphoproliferative Syndrome) (6)
- presence of deficiencies in the cell cycle check-points for which the cell cycle, fundamental to allow a correct repair of the damage, is missing (Cartilage-hair hypoplasia) (8)
- defects in the cytokinesis which, by hindering cell division, lead to the formation of genetically unstable tetraploid cells (Neutropenia X linked and Wiskott-Aldrich syndrome) (9).

Since the PID are numerous, the mechanisms that explain the increased susceptibility of these patients to the development of tumors are multiple and often different in the various pathologies. Certainly, the most important process, which is common to many immune defects with a predisposition to carcinogenesis, is represented by the reduction of cell-mediated immunosurveillance (as occurs in combined immunodeficiencies). This can be explained by the fact that this component of the immune system plays a fundamental role in protecting against tumors. In support of this thesis, there is then the evidence that the most frequent tumors found in patients with PID are represented by lymphomas, neoplasms that afflict the cells of the specific immunity. Finally, in many cases [for example in Omenn Syndrome or in WAS (6)] the presence of a predominance of type 2 cytokines and a reduction of type 1 cytokines, such as INF-α, was found to be essential for the control of the development of tumoral pathologies, in particular those of lymphoproliferative type. Instead, infections, whether *de novo*, reactivated or chronic, can play a decisive role in the genesis of both blood tumors and solid tumors [>20% of carcinomas in patients with PID are induced by infections (4)], through two mechanisms main: activation of oncogenic viruses and chronic antigenic stimulation. As regards the first case, an example is represented by the Epstein Barr virus (EBV), which is the most frequently found infectious agent in these tumors, especially in type B lymphomas, but also in those of type T. In particular, according to the ICR data, it is found in 30–60% of patients with lymphoproliferative disorders and PID. This condition is the result of a reduction, caused by the underlying immunodeficiency, of EBV-specific CD8 T cells. Therefore, PIDs with T cell defects are those in which the greatest susceptibility to the development of EBV+ (10) lymphomas is expected to be found. Furthermore, another effect favoring the development of tumors depends on the EBV's ability to express genes that inhibit cell-mediated immunity. In this way the virus becomes immortal within B cells, which proliferate uncontrollably under the viral stimulus, acquiring mutations due to loss of heterozygosity and/or cytogenetic rearrangements (6).

Instead, as regards the mechanism of chronic antigenic stimulation, an example is represented by Helicobacter pylori (HP) infections, which is implicated in the genesis of gastric carcinoma and gastric MALT lymphoma. The increased risk of infections from this microorganism in patients with PID [in particular, it is frequently found in common variable immunodeficiency (CVID)] suggests a genesis due to a reduction in the production of gastric IgA and hydrochloric acid, which facilitates the colonization of HP. This microorganism promotes carcinogenesis by establishing chronic inflammation by stimulating the local production of cytokines, which alter the adhesive properties of the surface of the gastric mucosa and promote ectopic proliferation of lymphoid tissue (6). Finally, in some cases the tumor arises as a result of cellular genomic instability, which favors the acquisition of numerous genetic mutations. In fact, some immunodeficiencies are characterized by the presence of defects in DNA repair mechanisms (as in the AT, where the ATM gene mutation reduces the functionality of the homonymous protein, which normally acts as a DNA double helix damage sensor and activates different repair mechanisms of these ones). In these cases, the DNA is continually exposed to potentially harmful insults, both extrinsic (for example from radiation of the environment), and intrinsic (for example from products of metabolism), which, not being able to be repaired, necessarily cause the appearance of mutations which favor cell degeneration toward neoplastic forms. In other cases, the acquisition of mutations is favored by the presence of apoptosis deficiency, which prevent the cell from meeting a programmed natural death following the appearance of DNA damage (as occurs in ALPS, for FAS, FAS mutations ligand or caspase 8) (6).

Furthermore, in some immunodeficiencies the acquisition of mutations is favored by the presence of defects in the cell cycle check-points (as occurs in cartilage-hair hypoplasia, which is caused by mutations in the RMRP gene, which determine an alteration of the cleavage of the Ciclin B2 mRNA, fundamental for the cell cycle control function). These mechanisms are normally able to detect possible DNA damage and stop the cell cycle for the time necessary to guarantee a correct repair (6), which, instead, will be absent in these pathologies. Finally, alterations in cytokinesis, as occurs in the XLN (Neutropenia X-linked) and probably also in the WAS, determine the formation of genetically unstable tetraploid cells, which facilitate the development of aneuplode tumor cells. Thus, as has been pointed out above, the mechanisms that explain the occurrence of tumors in patients with PID are numerous. Since many steps are needed to determine the appearance of a tumor, it is likely that several processes are simultaneously present in the same patient (7). For example, data from the ICR show that often brothers with the same immunodeficiency develop tumors of the same histotype. This could indeed be explained by the coexistence of multiple common favoring factors, such as exposure to the same carcinogens and having inherited greater susceptibility to the malignant transformation of specific clones and/or selective inability to destroy certain neoplastic cells (11).

## Methods

We decided to carry out a retrospective analysis at the Brescia Children's Hospital (Italy) reporting the incidence of tumors among 690 patients with a diagnosis of PID, diagnosed between 1990 and 2017 in Brescia. The study was approved by the Ethical Committee of the Spedali Civili of Brescia, president Aldo Maria Roccaro.

The study has the main purpose of reporting the experience of an Italian Pediatric Children's Hospital with patients suffering from PID who developed an oncological pathology during their lifetime, trying to report their characterizing elements.

## Results and Discussion

**Figure 1:**
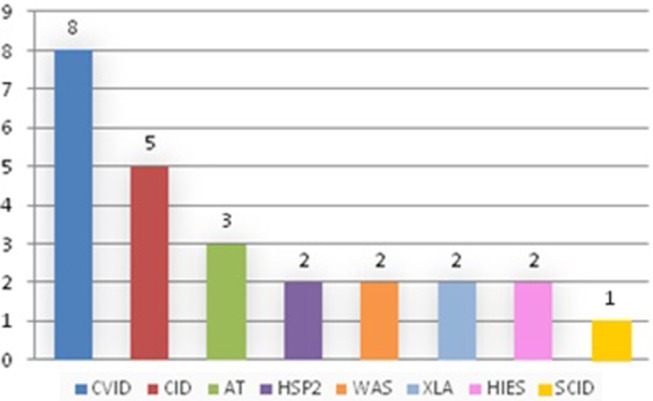
Patients with PID.

**Figure 2:**
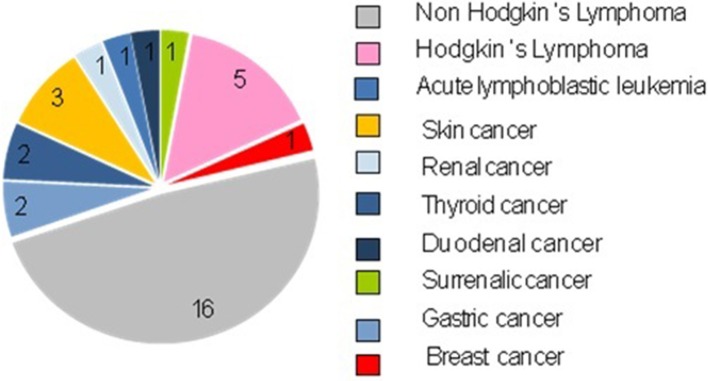
Types of tumors present in our records.

Out of 690 patients, 25 patients (3.6%) developed 33 tumors. They were treated differently depending on the tumor and the immunodeficiency: in particular, for solid tumors surgery was performed, with or without chemotherapy, while for hematological tumors, the treatment was based on National or International therapeutic protocols (Figures 1, 2).

In the literature it is reported that 4–25% of patients with PID develop a neoplasm (12), but we must consider that it is difficult to establish the exact incidence of tumors that arise in these patients. First of all, this is due to the fact that PID are rare diseases that make it difficult to collect an amount of data big enough to allow for results to be truly representative of the phenomenon. Secondly, it is possible that in many cancer patients no underlying PID is diagnosed, especially when this presents a mild clinical manifestation and/or a late onset, leading to an inevitable underestimation of the cases (13). Furthermore, the incidence of cancer often presents regional divergences, which are probably the consequence of a different distribution of the various PID subtypes in the population, of the variability of the exposure to pathogens that promote carcinogenesis and of the high frequency of genetic variants that influence the individual's susceptibility to the development of tumors (14), making the comparison of data obtained from studies on different populations even more complex.

**Figure 3:**
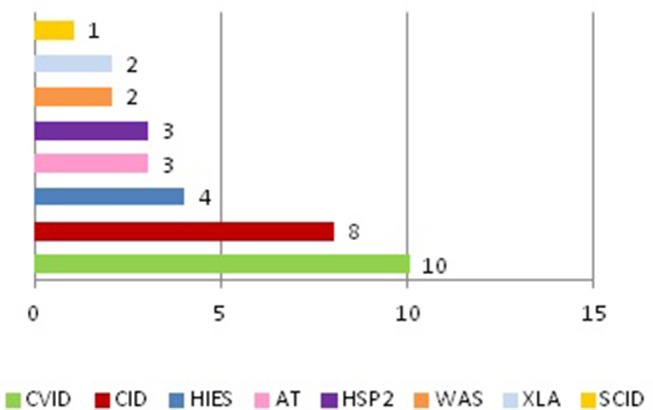
Cases of tumors in patients with PID.

We discovered that tumors appeared only in some of the primitive immunodeficiencies present in our Center (Figure 3). Indeed of the 25 affected patients, 8 patients suffered from CVID, 5 from combined immunodeficiency (CID), 3 from Ataxia-telangectasia (AT), 2 from Hermanksy-Pudlak type 2 (HSP2), 2 from agammaglobulinemia X-linked (XLA), 2 from Wiskott-Aldrich syndrome (WAS), 2 from Hyper IgE syndrome (HIES), 1 from Severe combined immunodeficiency (SCID) suggesting that not all these immunodeficiencies present an equal susceptibility to the development of cancer (15). In fact, no case has been identified among patients with Chronic granulomatous disease (CGD) or IgA deficiency (IgAD), confirming the data present in the literature, in which only a few cases of cancer are reported in these pathologies (10, 16, 17). It is also important to say that SCID and WAS are currently treated early with bone marrow transplantation, which has the advantage of ensuring a complete reconstitution of the functionality of the immune system and which could justify the small number of cases of cancer found in our records (1 out of 123 patients with SCID and 2 out of 52 patients with WAS). Moreover, in recent years the tendency of our Center is to rapidly perform a bone marrow transplant even in patients with CID, since experience has shown that executing of this procedure only after the development of a hematological tumor may be insufficient in the control of these neoplasms (two patients presented with tumor relapse despite bone marrow transplant). Therefore, in the next few years we expect a reduction in cancer cases in these patients too.

Another interesting finding is about the two patients with HSP2, who are two siblings, who both developed Hodgkin's lymphoma, a neoplasm that had never previously been identified in this immunodeficiency. In fact, before now, this syndrome was known only to determine a predisposition to the development of skin tumors (in our case a Dermatofibroma was detected) (18).

Among the cases we analyzed, patients who developed cancer were mostly male: 17 males (68%) and 8 females (32%). These data appear to be discordant with what emerges from the major studies in the literature. In fact, in two of these a distribution of cases of similar cancer was found in males and females (16, 17), while in one of them the females appeared more affected than males (17). In the latter case, if the individual tumors are analyzed, however, it is clear that the most common (non-Hodgkin's lymphoma, leukemia, and stomach tumors) have been presented with equal frequency in males and females and that the excess of cases present in the female subjects is attributable to the high number of cases of breast cancer (20% of all cancers). Instead, the most frequent occurrence of cases in males, which was highlighted in the present study, is probably the consequence of the fact that the cohort of patients with PID in our center is characterized by a clear male prevalence (435 male patients out of 690 total, 63%), which can at least partially be explained by the fact that many of these diseases (XLA, WAS, XSCID, and 60% of CGD cases) have an X-linked transmission and, therefore, only affect males. These divergences explain the heterogeneity of the distribution of immunodeficiencies and tumors in the different populations and the consequent difficulty in studying the general characteristics of this phenomenon.

**Figure 4:**
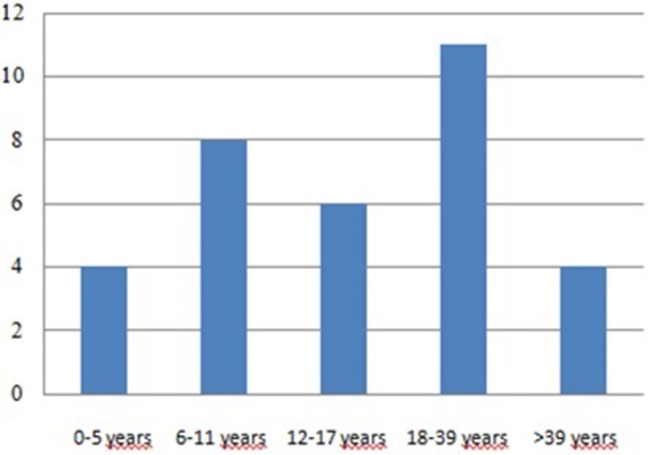
Age of tumor development.

The age at diagnosis ranged from 1 to 52 years, with a median age of 19.6 years. These data are similar to those derived from the ICR (Immunodeficiency Cancer Registry), where the average age was 20 years (19). Considering all 25 patients with tumors, 18 tumors occurred in 14 patients aged below 18 years, while 15 cancers occurred in 11 patients aged over 18 years old (Figure 4; Tables 1, 2). We have to consider that the age of onset of the various neoplasms appeared different related to the underlying immunodeficiency: in the CVID the diagnosis occurred at a mean age of 32.75 years and this is in line with what is found in the literature, which shows how CVID is an immunodeficiency that in itself is usually manifested in adulthood, as well as also a possible subsequent cancer (11). In the patients with SCID and CID the tumor arose at a mean age of 9 years which seems to conform to what is evident from the literature, in which the peak of onset is described between 0 and 10 years (11). In other PID the age of onset of the first neoplasia was very variable: in XLA at 1 and 37 years, in WAS at 4 and 27 years, in AT at 5, 14, and 29 years and in HSP2 at 8 and 10 years.

**Table 1:** Tumors in pediatric age (<18 years).

**Patients (*n* = 14)**	**Age**	**Tumors (*n* = 18)**
1 G.Z.	10	Linfoblastic NHL T
2 D.C.	10	Scleronodular Classic LH
3 N.C	4	NHL (nd)
5 F.R.	1	Acute Linfoblastic Leukemia
6 R.A.S.	7	Adrenal Leiomyosarcoma
	12	Diffuse large B-cell lymphoma
7 M.P.	11	Diffuse large B-cell lymphoma
10 O.B.A.	5	Diffuse large B-cell lymphoma
11 F.D.	14	Papillary thyroid cancer
13 N.M.	14	Diffuse large B-cell lymphoma
14 M.V.^*^	10	Nodular lymphocyte predominant Hodgkin lymphoma
15 G.V.^*^	8	Nodular lymphocyte predominant Hodgkin lymphoma
	11	Dermatofibroma
16 L.M.	5	Burkitt NHL
17 A.C.	10	Nodular sclerosis classical Hodgkin lymphoma
	17	Mycosis Fungoide
18 S.G.	12	Mixed cellularity classical Hodgkin lymphoma
	17	Diffuse large B-cell lymphoma

* *Brothers*.

**Table 2:** Tumors in adult age (>18 years).

**Patients (*n* = 11)**	**Age**	**Tumors (*n* = 15)**
4 G.B.	20	Papillary thyroid cancer
8 M.B.	37	Gastric carcinoma
9 G.C.	29	Diffuse large cells lymphoma
12 A.M.	43	Burkitt's lymphoma
19 F.C.	27	Plasmablastic lymphoma
20 L.C.	35	Diffuse large cells lymphoma
	37	Squamous cell carcinoma *in situ*
21 E.A.	22	MALT NHL
22 I.V.	35	Gastric NIN
23 E.V.	45	Clear cell renal cell carcinoma
	45	Duodenal GIST
24 E.L.	24	Anaplastic large cell lymphoma
	28	Basal cell carcinoma
	32	Diffuse large cells lymphoma
25 R.M.	52	Lobular carcinoma of the breast

We also evaluated the latency time between the diagnosis of PID and the appearance of the first tumor, which was very different from case to case (from 1 month to 29 years). For example, there was a short latency (in 5 patients <1 year) between the diagnosis of SCID/CID and the subsequent appearance of cancer. Indeed, in two cases of CID the tumor was diagnosed even before the underlying immunodeficiency, constituting the clinical manifestation of onset.

This finding has already been reported in the literature, in particular in those PIDs that present a delayed onset and/or a mild clinical manifestation (4). Therefore, the wide variability that was found did not allow to identify either an age or a time of homogeneous latency of presentation of the neoplasms. For this reason, screening investigations for the most frequent tumors in these patients should be performed early, immediately after the diagnosis of PID.

The presence of a marked predisposition to the development of neoplasms is suggested in our study by the number of tumors: 25 patients showed 33 tumors, of which 26 were early tumors, of which two were synchronous and one bilateral, 6 were second tumors, and 1 a third tumor.

Most of the tumors were malignant (30 cases, 90.9%), only 3 cases (9.1%) were non-invasive tumors (non-infiltrative intraepithelial neoplasia of the stomach, dermatofibroma, and 1 squamous cell carcinoma *in situ*).

The neoplasms were mostly hematological (22 cases, 66.67%): 21 cases of lymphomas (5 cases of Hodgkin's lymphomas and 16 cases of Non-Hodking's lymphomas), 1 case of Acute Linfoblastic Leukemia.

Non-Hodgkin's lymphomas were the most frequent tumors (16/33, 48.48%). We identified the different subtypes: 8 diffuse large cell lymphomas, 2 Burkitt lymphomas, 1 gastric MALT lymphoma, 1 lymphoblastic T cell lymphoma, 1 plasmablastic B cell lymphoma, 1 folliculotropic mycosis fungoides, 1 anaplastic large cells lymphoma, and 1 non-determined subtype.

These lymphomas presented peculiar characteristics: (1) frequent extranodal onset (12/15 cases); (2) Prevalence of B phenotype (12/15); (3) Majority of diffuse large cell Lymphoma B (8/15). These results also appeared to be aligned with those present in the literature and this is fundamental, since it is thanks to the knowledge of the common characteristics of these tumors that it is possible to make a diagnostic plan aimed at their early identification (15).

There was a minority of solid tumors (11 cases, 33.33%), which appeared to be very heterogeneous both by histotype and by localization: 3 cases of skin tumors (1 dermatofibroma, 1 squamous cell carcinoma *in situ* and 1 basalioma), 2 cases of thyroid tumors (papillary thyroid carcinoma), 2 gastric tumors (1 diffuse gastric carcinoma, and 1 non-infiltrative intraepithelial neoplasia of the stomach), 1 case of surrenalic tumor (leiomyosarcoma), 1 case of duodenal tumor (gastrointestinal stromal tumor, GIST), 1 case of breast cancer (lobular breast cancer), and 1 case of renal tumor (clear cell renal cell carcinoma).

Regarding specifically the tumors that appeared at a pediatric age, it was interesting to note that many were atypical for this age: Dermatofibroma, Fungal Mycosis, Papillary thyroid carcinoma, and Leiomyosarcoma. In contrast, the most common types of solid tumors of infancy were not found, such as Neuroblastoma, Wilms Tumor, and Rhabdomyosarcoma, as previously reported in the literature (11). This condition suggests that the immune system has a decisive role in controlling the development of only some types of cancer and to date the cause of this phenomenon has not yet been understood Table 3.

**Table 3:** Tumor in our patients.

**PID**	**Number of patients with PID**	**Number of patients with PID and tumor**	**Number of tumors**	**Type of tumor**		
				**1^**°**^ tumor**	**2^**°**^ tumor**	**3^**°**^ tumor**
CVID	74	8	10	1 HL	1 Squamous cell carcinoma *in situ*	
				1 Papillary thyroid cancer		
				3 NHL: (1 MALT, 1 Burkitt e 1 diffuse large B cell lymphoma)		
				1 Lobular carcinoma of the breast		
				1 Gastric NIN		
				1 Clear cell renal cell carcinoma		
				1 Duodena GIST		
CID	29	5	8	2 HL	3 NHL:	
				2 NHL: (1 linfoblastic T; 1 diffuse large B cell lymphoma)	(2 Diffuse large B cell lymphoma; 1 Mycosis fungoide)	
				1 Adrenal leiomyosarcoma		
SCID	123	1	1	1 Diffuse large cells lymphoma		
AT	19	3	3	3 NHL: (2 diffuse large B cell lymphomas; 1 Burkitt)		
XLA	46	2	2	1 Acute linfobastic leukemia		
				1 Gastric carcinoma		
HSP2	2	2	3	2 HL	1Dermatofibroma	
HIES	13	2	4	Papillary thyroid cancer	1 Basalioma	1 Diffuse large cells lymphoma
				1 Anaplastic large cells lymphoma		
WAS	52	2	2	2 NHL: (1 Plasmablastic lymphoma 1 nd)		

**Figure 5:**
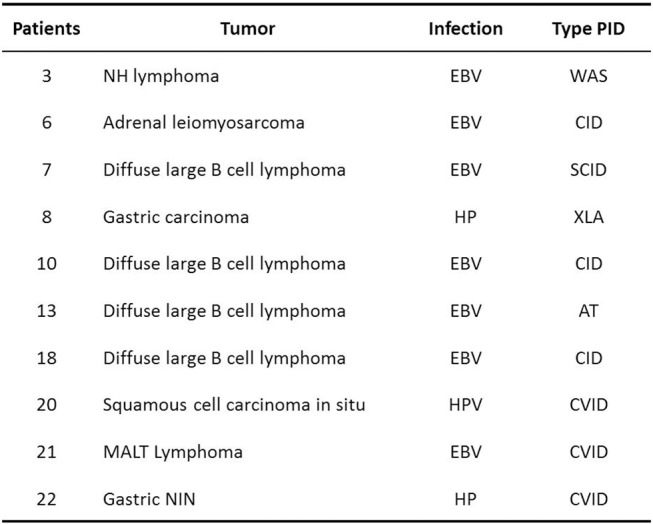
Tumors and infection.

Moreover, in our study in various situations, the anatomo-pathological evaluation allowed to identify microbial agents in the tumor microenvironment: EBV, HP, and HPV, confirming their role in the etiopathogenesis of these neoplasms. Therefore, it is important to identify and treat these infections early, with the aim of preventing related cancers Figure 5.

In the period considered, 12 patients died (48%) in total and the tumor was the leading cause of death (7 cases). In particular, in our experience among patients who developed cancer at pediatric age, 7/11 patients (63.6%) survived 5 years after diagnosis, a number that appears lower than the general population of patients with cancer of the same age, in which the 5-year survival is 82% (20). This discrepancy could be due to the appearance in these patients of rapidly progressive tumors, as confirmed by our study in which, in pediatric patients who died for the spreading of the tumor, death occurred in all cases a short time after the diagnosis (from 3 to 13 months). This condition does not seem to be due to the presence of tumors that are more resistant to treatment, but it is more likely the consequence of inadequate therapeutic management. In fact, in the absence of randomized clinical trials performed on these patients, the currently available chemotherapy treatment does not differ from that of immunocompetent patients, except for an individual modulation of the chemotherapeutic dosage and for the execution of a tight anti-infective prophylaxis. Despite the application of these measures, our experience has often shown a non-optimal response, as shown by the reduced survival, as well as the appearance of disease progression during the treatment of chemotherapy in 3 cases and relapses in 6 patients, all affected by lymphoma. As far as solid tumors are concerned, they have all been successfully treated and relapse has not occurred. The only exception was the case of gastric adenocarcinoma, which however appeared already metastatic at onset, to the point of contraindicating any treatment.

## Conclusions

Our descriptive study certainly has the advantage of a very large number of cases (690) for a single Center considering the rarity of these diseases, a very long observation period (27 years) and good expertise in PID. The limits are instead represented by the difficulty of finding all the blood tests of older patients, above all because in many cases some immunological tests were not yet carried out. Furthermore, such a long observation time helps to highlight the importance of monitoring patients with PID in order to recognize and treat tumors early.

Therefore, the correct management of tumors that arise in patients with PID still represents a challenge in the pediatric field. For this reason now it is mandatory to collect in a unique international registry the cases of malignancies in PID that could lead to a better understanding of the etiopathogenesis and of the biological and clinical characteristics of these tumors, with the aim of defining adequate preventive measures and guaranteeing an early diagnosis which also creating a shared and specific therapeutic strategy, with the prospect of obtaining a better prognosis for these patients.

## Data Availability

The datasets generated for this study are available on request to the corresponding author.

## Author Contributions

FP, RS, ES, and LN contributed conception and design of the study. AS organized the database. AL was involved in the manipulation of stem cells of transplant patients and has coordinated the activities of the stem cell laboratory. MM performed the statistical analysis, wrote the first draft of the manuscript and sections of the manuscript. All authors contributed to manuscript revision, read, and approved the submitted version.

### Conflict of Interest Statement

The authors declare that the research was conducted in the absence of any commercial or financial relationships that could be construed as a potential conflict of interest.

## Footnotes

^1^Document drawn up by AIEOP (Italian association of pediatric oncohematology) https://www.aieop.org/web/famiglie/schede-malattia/immunodeficienze/
